# Melatonin and the Dental Pulp: A Scoping Review

**DOI:** 10.1111/iej.70081

**Published:** 2025-12-09

**Authors:** Jasmin Schäfer, Konrad Kleszczynski, Edgar Schäfer

**Affiliations:** ^1^ Central Interdisciplinary Ambulance in the School of Dentistry University of Münster Münster Germany; ^2^ Department of Dermatology and Venerology University of Münster Münster Germany

**Keywords:** cell proliferation, cell senescence, dental pulp stem cells, inflammation, odontogenic differentiation, oxidative stress

## Abstract

**Background:**

In general medicine, melatonin is known to enhance wound healing and promote stem cell differentiation. Its potential relevance in endodontics, however, remains underexplored.

**Objectives:**

This scoping review aimed to systematically assess the available evidence on the effects of melatonin (a) on dental pulp tissue and (b) on human dental pulp stem cells (hDPSCs), particularly regarding cell proliferation and differentiation with regard to endodontics.

**Methodology:**

A comprehensive literature search was conducted in PubMed, Clarivate Analytics' Web of Science and Scopus from inception to July 1, 2025, using Medical Subject Headings (MeSH terms) and supplemented by hand searching and screening of major subject journals.

**Results:**

The initial search yielded 252 records, with one additional record identified through citation mining and relevant journal screening. A total of 22 studies met the inclusion criteria: 11 investigated melatonin's effect on dental pulp tissue regarding anti‐inflammatory properties, treatment of pulpitis, wound healing and pulp capping and 11 examined its impact on hDPSCs in terms of cell proliferation and differentiation.

**Discussion:**

The limited evidence obtained from laboratory and animal studies suggests a dose‐ and time‐dependent influence of melatonin, though evidence is insufficient to establish optimal concentrations.

**Conclusions:**

(a) Melatonin demonstrates anti‐inflammatory, antioxidant and anti‐fibrinolytic effects on dental pulp tissue. (b) Melatonin has potential as a stem cell modulator by promoting odontogenic differentiation and may improve migration and proliferation of hDPSCs.

## 1 Introduction

Melatonin (N‐acetyl‐5‐methoxytryptamine) is a bioactive indoleamine primarily synthesised and secreted by the pineal gland, with additional extra‐pineal production in various tissues such as the retina, gastrointestinal tract and skin (Andronachi and Popescu 2025; Slominski et al. 2025). Traditionally recognised for its role in regulating circadian rhythms and sleep‐wake cycles, melatonin has garnered significant attention for its multifaceted biological activities beyond its chronobiotic functions (Ahmad 2023). It exhibits potent immunomodulatory properties, influencing both innate and adaptive immune responses, including modulation of interleukin synthesis (e.g., IL‐2 and IL‐6) by mononuclear cells, which affects immune cell proliferation and cytokine production (Joseph and Kumar 2024). Melatonin also interacts with immune cells through high‐affinity melatonin receptors MT1 and MT2 to regulate immune functions (Calvo and Maldonado 2025).

In addition to immunomodulation, melatonin serves as a powerful antioxidant by scavenging reactive oxygen and nitrogen species and upregulating endogenous antioxidant enzymes such as superoxide dismutase and catalase (Ahmad 2023; Kołodziejska and Łukaszewicz‐Zając 2025). These properties help mitigate oxidative stress‐related tissue damage. Melatonin also exerts anti‐inflammatory effects by modulating inflammatory pathways, reducing pro‐inflammatory cytokine production and inhibiting inflammatory cell infiltration (Joseph and Kumar 2024; Slominski et al. 2025). Its anti‐apoptotic effects are attributed to stabilisation of mitochondrial function, reduction of mitochondrial reactive oxygen species and activation of pro‐survival signalling pathways, contributing to protection against various stress‐induced cell deaths (Slominski et al. 2025). Melatonin further demonstrates anti‐ageing properties through regulation of cellular senescence, DNA repair and mitochondrial maintenance (Yousefi and Khodadadi 2024; Slominski et al. 2025).

Exogenous melatonin generally has a short elimination half‐life of about 30–60 min, though formulation and route can extend its effective duration of action up to a few hours (Fourtillan et al. 2001). For oral immediate‐release melatonin, the half‐life is about 30–50 min; it is rapidly absorbed, with peaks in blood of about 30–60 min after ingestion, and thereafter declines quickly. Sublingual or transdermal forms have a similar elimination half‐life, but with different absorption kinetics (faster onset for sublingual; slower, more stable levels for transdermal) (Martínez et al. 2025).

Recent studies highlight the role of melatonin in tissue regeneration and stem cell biology. It promotes proliferation and differentiation of mesenchymal and neural stem cells under oxidative stress conditions and influences angiogenesis, a key process in tissue repair and regeneration (Navid and Sadeghi 2024; Kulka‐Kamińska et al. 2025; Rashidi and Ghaffari 2025). These multifaceted biological activities underscore melatonin's therapeutic potential in conditions characterised by oxidative stress and inflammation, including neurodegenerative diseases, cardiovascular disorders and chronic inflammatory conditions (Ahmad 2023; Kołodziejska and Łukaszewicz‐Zając 2025). Moreover, its effects on stem cell regulation and tissue repair suggest potential applications in regenerative medicine and tissue engineering (Navid and Sadeghi 2024; Rashidi and Ghaffari 2025; Slominski et al. 2025). Overall, melatonin's diverse roles in immunomodulation, antioxidation, anti‐inflammation, anti‐apoptosis, tissue proliferation, stem cell regulation and anti‐ageing make it a promising candidate for a wide range of clinical and translational applications (Andronachi and Popescu 2025; Böhm et al. 2025; Slominski et al. 2025).

Due to these unique properties, melatonin has attracted growing interest in dentistry (Gómez‐Moreno et al. 2010; Blasiak et al. 2011). It appears to protect oral tissues from oxidative stress–related diseases and exerts anti‐inflammatory effects, partly through inhibition of cyclooxygenase‐2 (COX‐2) (Gómez‐Moreno et al. 2010). Owing to its antioxidant properties, melatonin can shield pulp tissue from the cytotoxic and genotoxic effects of methacrylate monomers derived from resin‐based restorations (Blasiak et al. 2011) and can reduce the oxidative stress following tooth extraction (Cutando et al. 2007). Moreover, melatonin plays a role in periodontal wound healing by modulating fibroblast activity, stimulating type I collagen synthesis and promoting osteoblast differentiation and bone formation (Gómez‐Moreno et al. 2010; Vaseenon et al. 2021). In animal models, melatonin as a biomimetic agent stimulates bone growth around dental implants and facilitates their osteointegration (Gómez‐Moreno et al. 2010; Blasiak et al. 2011). Furthermore, melatonin's cytostatic properties suggest potential in oral cancer prevention and therapy (Bubenik et al. 1998; Gómez‐Moreno et al. 2010; Blasiak et al. 2011). For example, exogenous restoration of the melatonin receptor 1 A (MTNR1A) resulted in growth inhibition of oral squamous‐cell carcinoma (Nakamura et al. 2008).

Furthermore, some studies also pointed out that melatonin has some functions in the mineralisation of dental hard tissues. It contributes to ameloblastic differentiation as it stimulates enamel matrix protein transcription, synthesis and mineralisation of ameloblast lineage cells through the c‐Jun N‐terminal kinase 3‐β‐arrestin 1 (JNK3‐Arrb 1) pathway (Ren et al. 2021). Polymeric nanoparticles doped with melatonin applied in root canal‐treated teeth before obturation of the canals caused occlusion of dentinal tubules and mineral deposits onto the root canal wall dentine, resulting in reinforcement of the radicular dentine (Toledano et al. 2021). Besides, melatonin has been shown to promote odontoblastic differentiation during tooth development by upregulation of the malic enzyme 2 (ME2) (Zhang et al. 2023). Against this background, it is important to investigate whether, and to what extent, melatonin may exert relevant effects and hold therapeutic potential in endodontics. The only available narrative review on the effect of melatonin in wound healing of dental pulp tissue reported, on the basis of the results of nine available studies, some promising beneficial effects (Vaseenon et al. 2021).

This scoping review aims to expand current knowledge by identifying recent studies and systematically assessing the effects of melatonin on (i) dental pulp tissue, particularly in relation to inflammation, treatment of pulpitis, wound healing, pulp capping and (ii) human dental pulp stem cells (hDPSCs) with regard to cell proliferation and differentiation.

## 2 Methods

This scoping review was conducted following the PRISMA‐ScR framework for scoping reviews (Tricco et al. 2018) and as close as possible to the Preferred Reporting Items for Systematic Reviews and Meta‐Analyses (PRISMA) guidelines (Page et al. 2021) and relevant recommendations (Nagendrababu et al. 2020). The aim was to provide the best available evidence to answer the following questions: (i) What is the impact of melatonin on the pulp tissue? (ii) What is the impact of melatonin on hDPSCs?

Inclusion Criteria

Eligibility criteria were as follows:

1. In vitro, in vivo and clinical studies on the effect of melatonin on dental pulp tissue: anti‐inflammatory effects, pulpitis, wound healing and pulp capping.
2. In vitro, in vivo and clinical studies on the effect of melatonin on hDPSCs regarding cell proliferation and differentiation with regard to endodontic issues.

Exclusion Criteria

1. Narrative Reviews
2. Use of stem cells from the apical papilla
3. Use of hDPSCs for non‐endodontic issues
4. Effect of melatonin on apical periodontitis
5. Effect of melatonin on mineralisation of dental hard tissues (enamel, dentine)
6. Use of hDPSCs for non‐endodontic purposes
7. Studies published in languages other than English

### 2.1 Information Sources and Search Strategy

The literature search was performed on the 5th of December 2024 and was conducted in accordance with PRISMA‐S guidelines (Rethlefsen et al. 2021). The search process was rerun after finalisation of the final drafting of the manuscript on the 1st of July 2025 to ensure that studies published after the first literature search were also included. The following electronic databases were searched without date or status of the publication restrictions: PubMed (including MEDLINE), Clarivate Analytics' Web of Science and Scopus.

Medical Subject Headings (MeSH terms; https://www.ncbi.nlm.nih.gov/mesh) were selected for the initial search process. The MeSH term ‘Melatonin’ was annexed with the Boolean operator AND with the terms ‘dental pulp’ (MeSH), ‘pulpitis’ (MeSH), ‘dental pulp stem cells’ (MeSH), ‘pulp capping’ (MeSH), ‘endodontics’ (MeSH) and ‘root canal’ (MeSH). The full search strategy with the corresponding findings is summarised in Table 1. Furthermore, the most relevant dental journals (*International Endodontic Journal*, *Journal of Endodontics*, *Journal of Dental Research*, *Journal of Dentistry and Clinical Oral Investigations*) were also screened. Finally, the reference lists of all resulting articles were also screened for potentially relevant studies. The search results were imported into Mendeley (Mendeley Desktop, George Mason University, USA) for duplicate removal and further investigation.

**TABLE 1:** Adapted search strategies.

		PubMed	Scopus	Web of Science	Sum
‘melatonin’ (MeSH) AND	‘dental pulp’ (MeSH)	32	46	41	119
	‘pulpitis’ (MeSH)	6	8	7	21
	‘dental pulp stem cells’ (MeSH)	16	28	22	66
	‘pulp capping’ (MeSH)	1	1	1	3
	‘endodontics’ (MeSH)	16	2	2	20
	‘root canal’ (MeSH)	7	8	8	23
					252

### 2.2 Selection Process and Data Collection

**FIGURE 1:**
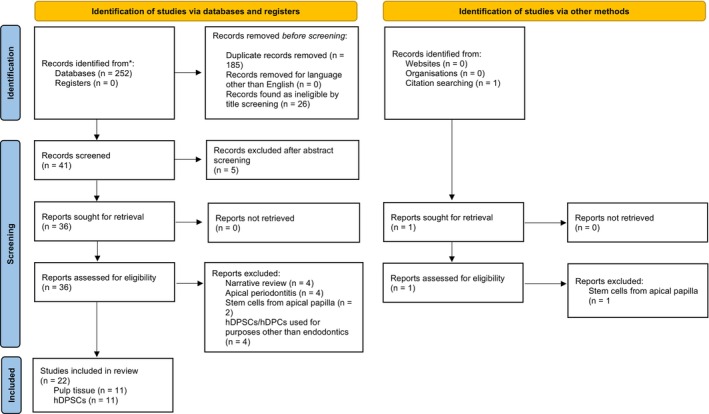
PRISMA 2020 flow diagram which included searches of databases, registers and other sources. *Detailed numbers of records identified from each database is given in Table 1; hDPC, human dental pulp cells; hDPSCs, human dental pulp stem cells.

Two reviewers (J.S. and E.S.) independently performed the initial screening of titles and abstracts based on the inclusion criteria. Articles not fulfilling the eligibility criteria were excluded, and for all remaining articles, the full texts were screened. Again, two independent reviewers (J.S. and E.S.) critically evaluated these full texts of studies. The obtained suggestions of eligible studies were compared, and any disagreements were resolved following discussion (Figure 1).

Data extraction was performed by the same reviewers using a pre‐established spreadsheet. The following details were extracted: name and country of authors, journal with year of publication, type of study design, interventions (agents, dose, duration), outcome measures, major findings and conclusions. All extracted data were stored in tables.

The inter‐reviewer reliability (percentage of agreement and kappa correlation coefficient) of the full‐text analysis was calculated and reported.

## 3 Results

### 3.1 Literature Search Process and Study Selection

Detailed information on the literature search sequence is given in the PRISMA flowchart (Figure 1). Initial searches revealed 252 hits across all the databases, and one study eligible for screening was found through citation mining and relevant journal screening, The language of the identified papers was limited to papers written in English and duplicates were removed, leaving 67 papers for title screening. Ineligible manuscripts were removed after title screening, and 42 manuscripts were subjected to abstract screening. Another five papers were excluded based on their abstracts, and thus 37 papers were sought for retrieval in full text. Out of these, narrative reviews (*n* = 4), studies on apical periodontitis (*n* = 4), the use of hDPSCs or hDPCs for purposes other than endodontics (*n* = 4) and studies using stem cells from the apical papilla (*n* = 3) were excluded. In total, 22 were included in the review, 11 assessing the impact of melatonin on dental pulp tissue regarding anti‐inflammatory effects, treatment of pulpitis, wound healing and pulp capping and 11 studying the effect of melatonin on hDPSCs with regard to cell proliferation and differentiation.

The inter‐reviewer reliability of the full text evaluation was high. The reviewers agreed in 94.6% of the manuscripts. The Kappa correlation coefficient for inter‐reviewer reliability was 0.888, indicating a very good strength of agreement between the reviewers.

### 3.2 Characteristics of the Included Studies

#### 3.2.1 Effect of Melatonin on Dental Pulp Tissue

In total, 11 studies were included: eight laboratory studies (Milosavljević et al. 2018; Deng et al. 2019; Kantrong et al. 2020; Chang et al. 2022; Barać et al. 2023; Ilić et al. 2023; Yu et al. 2024), three animal studies (Doğan et al. 2017; Guerrero‐Gironés et al. 2020; Kermeoğlu et al. 2021; Chang et al. 2025), and one study that conducted both laboratory and animal investigations (Li et al. 2015). Characteristics and results of the included studies are summarised in detail in Tables 2 and 3. Cell types used for investigation were hDPCs (Milosavljević et al. 2018; Deng et al. 2019; Kantrong et al. 2020; Chang et al. 2022, 2025; Barać et al. 2023; Ilić et al. 2023) and mouse preodontoblasts (mDDPC6T) (Yu et al. 2024). In three laboratory studies, hDPCs were obtained from both healthy and type 2 diabetic patients (Li et al. 2015; Milosavljević et al. 2018; Barać et al. 2023; Ilić et al. 2023). Regarding the animal studies, one study investigated the protective effect of melatonin against exogenous noxa (ELF‐EMF: extremely low frequency electric and magnetic field) (Doğan et al. 2017), one assessed the suitability of melatonin as an agent for direct pulp capping (Guerrero‐Gironés et al. 2020) and one analysed the trunk blood and pulp tissue of rats with and without acute pulpitis (Kermeoğlu et al. 2021).

**TABLE 2:** Characteristics and results of the included laboratory studies on the effect of melatonin on dental pulp tissue regarding anti‐inflammatory effects, treatment of pulpitis, wound healing and pulp capping.

Studies	Study type & model	Intervention	Main findings	Conclusions	Main effects of Mel
Li et al. (2015)	Laboratory study: hDPSCs from healthy patients (18–22 years) (*n* = 8)	Mel 1.0 mM/L; 1, 6 h	Inhibition of gene expression of pro‐inflammatory cytokines: TNF‐α ↓ IL‐1β ↓ Modulation of TLR4/NF‐ĸB signalling TLR4 ↓ I‐ĸB ↑	Mel can act directly on pulp cells by modulating TLR4/NF‐ĸB signalling	Anti‐inflammatory
Doğan et al. (2017)	Animal study: 56 adult Wistar rats were exposed to ELF‐EMF for 8 h/day for 26 days (*n* = 8 per group)	Mel 10 mg/kg/day (orally); 26, 52 days	ELF‐EMF caused odontoblast degeneration, increased inflammatory cell infiltration, dilatation in blood vessels and haemorrhage Mel for 26 and 52 days odontoblast activity ↑ proliferation of fibroblasts ↑ induction of neo‐angiogenesis ↑	Mel can protect the pulp tissue from adverse effects caused by ELF‐EMF	Pulp protection against exogenous noxa (ELF‐EMF)
Milosavljević et al. (2018)	Laboratory study: hDPCs from 16 healthy and 16 type 2 diabetic patients (50–70 years)	Mel 0.1 and 1.0 mM/L; 24 h	Both Mel concentrations showed no cytotoxicity In diabetic dental pulp tissue: Mel ↓ 1.0 mM Mel decreased hyperglycaemia‐induced increases of iNOS and increased SOD activity in normoglycemic levels iNOS and p300 protein expression levels showed a strong positive correlation under hyperglycaemia	Under hyperglycaemic conditions, Mel normalises iNOS and SOD activity levels Under hyperglycaemic conditions, Mel has antioxidant and protective effects on pulp cells	Antioxidant
Deng et al. (2019)	Laboratory study: hDPCs from 13 patients (18–25 years)	Mel 10^−12^, 10^−10^, 10^−8^ M/L; 24 h H_2_O_2_ 250, 500, 1000 μM/L	H_2_O_2_ decreased the viability of hDPCs in a concentration‐dependent manner Mel alone reduced viability by 16%–20% Mel 10^−8^ M/L showed the most obvious effect Treatment of H_2_O_2_‐exposed hDPCs with Mel: ratio of apoptotic cells ↑ ROS ↑ Mitochondrial membrane potential (Δᴪm) ↓	At physiological concentrations, Mel can enhance H_2_O_2_‐induced apoptosis, increase H_2_O_2_‐mediated ROS production and Δᴪm loss	No antioxidant effect
Guerrero‐Gironés et al. (2020)	Animal study: Male rats (*n* = 16; *n* = 16 teeth/group)	Direct pulp capping; 30 days Group I: MTA Group II: Mel 5 mg; topical Group III: MTA + Mel 10 mg/100 mL of drinking water Group IV: Mel 5 mg and 245 mg cornstarch + distilled water (topical) + Mel 10 mg/100 mL of drinking water	Comparison of groups I–IV: Degree of pulp inflammation ↔ Degree of pulp necrosis ↔ Dentinal bridge formation ↔ Odontoblastic layer ↔ Pulp fibrosis ↔ TBARS in blood, kidney, liver ↔	Effect of Mel regarding direct pulp capping was similar to that of MTA	Agent for pulp capping
Kantrong et al. (2020)	Laboratory study: Phase I: healthy human dental pulp tissue (*n* = 3) Phase II: human dental pulp tissue; healthy *n* = 12, pulpitis *n* = 6 Phase III: hDPCs *n* = 3 Age of patients not given	Phase III: Mel 10^−3^, 10^−6^, 10^−9^, 10^−12^ M; 3, 24 h LPS 20 μg/mL; 24 h	Phase I: Mel expression within the odontoblastic zone, cell‐rich zone and in the connective tissue Phase II: healthy: strong MT1 and MT2 expression pulpitis: MT1 expression ↔; MT2 expression ↓ Phase III: LPS + Mel 10^−3^ M IL‐1β mRNA ↓ COX‐1 mRNA ↔ COX‐2 mRNA ↑	Dental pulp tissue expresses Mel with strong MT1 and MT2 expression Mel has selective antagonistic characteristics for LPS‐mediated inflammatory signal induction via COX‐2 and IL‐1β Mel might be beneficial for the treatment of pulp inflammation	Anti‐inflammatory
Kermeoğlu et al. (2021)	Animal study: Male and female rats (*n* = 32)	Group I: control, untreated rats, 24 h Group II: acute pulpitis, 24 h Group III: acute pulpitis + Mel 10 mg/kg, i.p., 24 h	Analysis of trunk blood and pulp tissue Comparison of groups I and II: TNF‐α ↑ IL‐1β ↑ MMP‐1 ↑ MMP‐2 ↑ Comparison of groups II and III: TNF‐α ↓ IL‐1β ↓ MMP‐1 ↓ MMP‐2 ↓	Mel shows protective effects on acute pulpitis Mel might be beneficial for the treatment of pulp inflammation	Anti‐inflammatory
Chang et al. (2022)	Laboratory study: hDPCs from young donors (caries‐ and periodontitis‐free premolars) (sample size and age not given)	hDPCs were exposed to IL‐1β or Mel (50, 100, 200 μg/mL) alone and to IL‐1β with/without Mel pretreatment	Effects of Mel on the expression/production of: uPA ↓ uPAR/suPAR ↑ PAI‐1 ↑ Effects of Mel on IL‐1β‐induced changes: uPA mRNA ↓ uPAR ↑ PAI‐1 mRNA ↑	Mel exerts an anti‐fibrinolytic effect on IL‐1β‐induced changes Mel might be beneficial for the prevention and treatment of pulp inflammation	Anti‐fibrinolytic anti‐inflammatory
Barać et al. (2023)	Laboratory study: hDPCs from 15 healthy and 15 type 2 diabetic patients (age not given)	Mel 0.1 mM, 1.0 mM; 24 h	Diabetic patients: GDNF ↑ MEL ↓ iNOS activity ↑ SOD activity ↑ Normoglycaemic hDPCs + Mel (0.1 mM): GDNF ↑ SOD ↑ Hyperglycaemic hDPCs + Mel (1.0 mM): GDNF ↓ SOD ↓ iNOS ↓	Mel induces the stress‐protective mechanism in hyperglycaemic hDPCs Mel can be beneficial for diabetes‐associated disturbances in pulp tissue	Antioxidant
Ilić et al. (2023)	Laboratory study: hDPCs from 7 healthy and 6 type 2 diabetic patients (55–65 years)	hDPCs were exposed to HEMA (5 mM) and/or CQ (1 mM) in the absence and presence of Mel (0.1 mM, 1.0 mM; 24 h)	Both Mel concentrations had no effect on cell viability Diabetic patients: MEL ↓ iNOS ↑ Effects of Mel (0.1 mM) on HEMA‐ and CQ‐induced changes: HMOX1 mRNA ↓ NOX4 mRNA ↓ SOD activity ↓ iNOS ↑ Effects of Mel (1.0 mM) on HEMA‐ and CQ‐induced changes: BCL‐2 expression ↓ HMOX1 mRNA ↑ NOX4 mRNA ↑ SOD activity ↓ iNOS ↑	Mel counteracts iNOS‐mediated inflammatory and stress effects in HEMA‐ and CQ‐treated hDPCs	Anti‐inflammatory Antioxidant Pulp protection against exogenous noxa (resin monomer)
Yu et al. (2024)	Laboratory study: Mouse preodontoblast cells (mDPC6T)	TEGDMA (2 mM) Mel (50, 100, 150, 200 μM); pretreatment for 2 h before TEGDMA exposure	Effects of Mel (0.1 mM) on TEGDMA‐induced apoptosis: apoptosis ↓ mtROS ↓ MMP ↓ ATP ↓	Mel protects preodontoblast cells against TEGDMA‐induced apoptosis via the JNK/MAPK signalling pathway Mel could be useful for the prevention of resin monomer‐induced pulp alterations	Anti‐apoptotic Pulp protection against exogenous noxa (resin monomer)
Chang et al. (2025)	Laboratory study: hDPCs; donor and age not given	Mel 50, 100 μm/mL	TriDAP stimulated cPLA2 and COX‐2 expression, and PGE_2_ and PGE_2α_ secretion in hDPCs Effects of Mel (50 and 100 μm/mL): PGE_2_ ↓ (near 100% reduction) PGE_2α_ ↓ (near 100% reduction)	Mel may inhibit TriDAP‐induced prostaglandin production in the dental pulp Mel can be used to control bacterial pathogen‐induced pulpal inflammation	Anti‐inflammatory

**TABLE 3:** Characteristics and results of the included animal studies on the effect of melatonin on dental pulp tissue regarding anti‐inflammatory effects, treatment of pulpitis, wound healing and pulp capping.

Studies	Animals and teeth used	Intervention	Main findings	Conclusions and critical appraisal	Main effects of Mel
Li et al. (2015)	Pulp tissue of Sprague Dawley rats with acute pulpitis (*n* = 32) Upper Molars	Mel 10 mg/kg/i.p.; 1, 2, 5 days	IL‐18 in serum ↓ TNF‐α in serum & pulp ↓ IL‐1β in serum & pulp ↓ TLR 4 mRNA ↓ NF‐ĸB mRNA ↓	Abdominal injection of Mel can significantly inhibit activation of pro‐inflammatory cytokine expression in the pulp tissue with AP Rats are less susceptible to pulpal infection than humans due to specific immune defence mechanisms. Thus, extrapolation of the findings to human pulp tissue is limited	Anti‐inflammatory
Doğan et al. (2017)	56 adult Wistar rats were exposed to ELF‐EMF for 8 h/day for 26 days (*n* = 8 per group) Anterior teeth	Mel 10 mg/kg/day (orally); 26, 52 days	ELF‐EMF caused odontoblast degeneration, increased inflammatory cell infiltration, dilatation in blood vessels and haemorrhage Mel for 26 and 52 days of odontoblast activity ↑ Proliferation of fibroblasts ↑ Induction of neo‐angiogenesis ↑	Mel can protect the pulp tissue from adverse effects caused by ELF‐EMF Rat incisors are continuously growing teeth; questionable whether the results are applicable to human pulp	Pulp protection against exogenous noxa (ELF‐EMF)
Guerrero‐Gironés et al. (2020)	Male Sprague Dawley rats (*n* = 16; *n* = 16 teeth/group) Molars	Direct pulp capping; 30 days Group I: MTA Group II: Mel 5 mg; topical Group III: MTA + Mel 10 mg/100 mL of drinking water Group IV: Mel 5 mg and 245 mg cornstarch + distilled water (topical) + Mel 10 mg/100 mL of drinking water	Comparison of groups I–IV: Degree of pulp inflammation ↔ Degree of pulp necrosis ↔ Dentinal bridge formation ↔ Odontoblastic layer ↔ Pulp fibrosis ↔ TBARS in blood, kidney, liver ↔	Effect of Mel regarding direct pulp capping was similar to that of MTA Considerable lack of standardisation as cavity depth and size were inconsistent leading to varying degrees of pulpal injury	Agent for pulp capping
Kermeoğlu et al. (2021)	Male and female Wistar rats (*n* = 32) Upper incisors	Group I: control, untreated rats, 24 h Group II: acute pulpitis, 24 h Group III: acute pulpitis + Mel 10 mg/kg, i.p., 24 h	Analysis of trunk blood and pulp tissue Comparison of groups I and II: TNF‐α ↑ IL‐1β ↑ MMP‐1 ↑ MMP‐2 ↑ Comparison of groups II and III: TNF‐α ↓ IL‐1β ↓ MMP‐1 ↓ MMP‐2 ↓	Mel shows protective effects on acute pulpitis Mel might be beneficial for the treatment of pulp inflammation Rats are less susceptible to pulpal infection than humans due to specific immune defence mechanisms. Thus, extrapolation of the findings to human pulp tissue is limited. Rat incisors are continuously growing teeth; questionable whether the results are applicable to human pulp	Anti‐inflammatory

Abbreviations: ATP, adenosine triphosphate; BCL‐2, B‐cell lymphoma‐2; COX‐1, cyclooxygenase 1; COX‐2, cyclooxygenase 2; cPLA2, cytosolic phospholipase A2; CQ, camphorquinone; ELF‐EMF, extremely low frequency electric and magnetic field; GDNF, glial cell line‐derived neurotropic factor; hDPCs, human dental pulp cells; hDPSC, human dental pulp stem cell; HEMA, 2‐hydroxylmethyl methacrylate; HMOX1, heme oxygenase‐1; i.p., intraperitoneal; I‐ĸB, I kappa B; IL‐18, interleukin 18; IL‐1β, interleukin 1 beta; iNOS, inducible NO synthase; JNK, c‐Jun N‐terminal kinase; LPS, lipopolysaccharide; MAPK, mitogen‐activated protein kinase; Mel, melatonin; MMP, mitochondrial membrane potential; MMP‐1, matrix metalloproteinase 1; MMP‐2, matrix metalloproteinase 2; MT1, melatonin receptor 1; MT2, melatonin receptor 2; MTA, mineral trioxide aggregate; mtROS, mitochondrial reactive oxygen species; NF‐ĸB, nuclear factor kappa light‐chain‐enhancer of activated B cells; NOX4, NADPH oxidase‐4; p300, histone acetyltransferase p300; PAI‐1, plasminogen activator inhibitor 1; PGE_2_, prostaglandin E_2_; ROS, reactive oxygen species; SOD, superoxide dismutase; suPAR, soluble urokinase‐type plasminogen activator receptor; TBARS, thiobarbituric acid reactive substances testing; TEGDMA, triethylene glycol dimethacrylate; TLR 4, toll‐like receptor 4; TNF‐α, tumour necrosis factor alpha; TriDAP, L‐Ala‐γ‐D‐Glu‐meso‐diaminopimelic acid; uPA, urokinase‐type plasminogen activator; uPAR, urokinase‐type plasminogen activator receptor.

Melatonin was added in physiological and pharmaceutical (micro/millimolar levels) concentrations, ranging from 10^−12^ to 10^−3^ M (Tables 2 and 3) in the laboratory studies, while in the animal studies 10 mg/kg was administered either intraperitoneally or with the drinking water. Melatonin at different concentrations (0.1 and 1.0 mM) did not show any cytotoxicity on hDPCs (Ilić et al. 2023; Milosavljević et al. 2018), while another study reported that physiological concentrations of melatonin (10^−12^, 10^−10^, 10^−8^ M) reduced the viability of hDPCs by 16%–20% (Deng et al. 2019).

**FIGURE 2:**
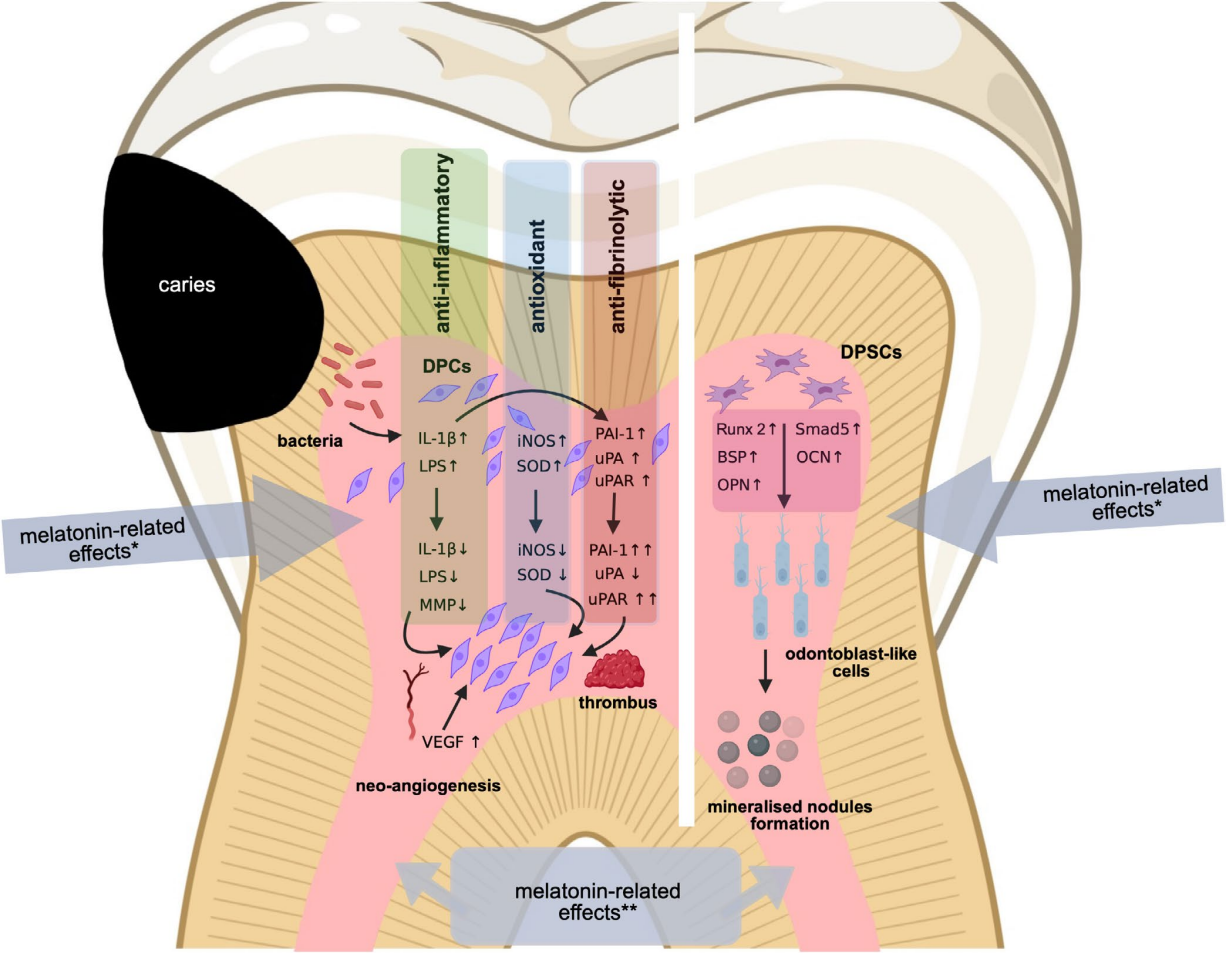
Schematic illustration of the potential melatonin‐related effects (*topical application; **systemic application) on dental pulp tissue (left side) and dental pulp stem cells (right side). The odontoblast icon represents odontoblast‐like cells and is used for visualisation purposes only. Created by BioRender.com.

Concurrently, six studies demonstrated that melatonin exerted an anti‐inflammatory effect on dental pulp tissue (Li et al. 2015; Kantrong et al. 2020; Kermeoğlu et al. 2021; Chang et al. 2022, 2025; Ilić et al. 2023) (Figure 2). In this context, Chang et al. (2022) showed that melatonin also exerted an anti‐fibrinolytic effect on IL‐1β‐induced tissue changes.

The antioxidant effect of melatonin on dental pulp tissue was investigated in four studies (Milosavljević et al. 2018; Deng et al. 2019; Barać et al. 2023; Ilić et al. 2023). Under hyperglycaemic conditions, melatonin normalised iNOS (inducible NO synthase) and SOD (superoxide dismutase) activity levels (Milosavljević et al. 2018; Barać et al. 2023; Ilić et al. 2023), showing that melatonin can be beneficial for diabetes‐associated disturbances in pulp tissue (Barać et al. 2023). However, at physiological concentrations, melatonin enhanced H_2_O_2_‐induced apoptosis, increased H_2_O_2_‐mediated reactive oxygen species (ROS) production and decreased mitochondrial membrane potential (Δᴪm) (Deng et al. 2019).

The ability of melatonin to protect the dental pulp against exogenous noxa, such as resin monomer (HEMA, TEGDMA) or ELF‐EMF, has been demonstrated in three studies (Doğan et al. 2017; Ilić et al. 2023; Yu et al. 2024).

One animal study showed that the histological outcome of direct pulp capping after topical application of melatonin (5 mg) or administration via drinking water (10 mg/100 mL) regarding the degree of pulp inflammation or pulp necrosis, the structure of the odontoblastic layer and the extent of pulp fibrosis was similar to that of the current gold standard (MTA: mineral trioxide aggregate) (Guerrero‐Gironés et al. 2020). This study also conducted an oxidative stress analysis using thiobarbituric acid reactive substances testing (TBARS) in blood, kidney and liver tissues. Again, no significant differences between the MTA group and the groups with melatonin treatment were obtained.

#### 3.2.2 Effect of Melatonin on Human Dental Pulp Stem Cells (hDPSCs)

Characteristics and results of the 11 included laboratory studies are summarised in detail in Table 4 (Maioli et al. 2016; Liu et al. 2017; Otero et al. 2017; Li et al. 2020; García‐Bernal et al. 2021; Mancinelli et al. 2021; Patil et al. 2022; Tumedei et al. 2022; Atila et al. 2023; Keskin et al. 2023; Zhang et al. 2024). In all but one study, hDPSCs were obtained from human donors, while in one study, dental pulp stem cells (DPSCs) were obtained from the American Culture Collection (Keskin et al. 2023). Melatonin was added in physiological and pharmaceutical (micro/millimolar levels) concentrations, ranging from 10^−12^ to 10^−4^ M (Table 4). Four studies assessed the odontogenic differentiation of hDPSCs in both basal growth and osteogenic (differentiation) medium (Liu et al. 2017; Li et al. 2020; Mancinelli et al. 2021; Tumedei et al. 2022) and consistently found superior results when using osteogenic medium. To assess the effect of melatonin on hDPSCs' mineralisation, alizarin red S staining (Maioli et al. 2016; Liu et al. 2017; Otero et al. 2017; Li et al. 2020; García‐Bernal et al. 2021), von Kossa staining (Patil et al. 2022), ALP staining (Atila et al. 2023; Zhang et al. 2024), determination of osteocalcin levels (Mancinelli et al. 2021; Tumedei et al. 2022) or intracellular calcium deposition measurements (Liu et al. 2017; Li et al. 2020; Atila et al. 2023) were used.

**TABLE 4:** Characteristics and results of the included studies on the effect of melatonin on dental pulp stem cells (DPSCs).

Studies	Cells and donors	Intervention	Main findings	Conclusions	Main effects of Mel
Maioli et al. (2016)	hDPSCs from 10 patients (15–26 years)	Medium A: Mel 0.01 M; 1, 3, 7, 14, 21 days Medium B: HA + BU + RA + Mel (0.01 M); 1, 3, 7, 14, 21 days	Expression of osteogenic specific genes: Medium A: VEGF A ↔ Runx2 ↑ (only at day 7) ZBTB16 ↔ NR4A3 ↔ STC1 ↔ OCN ↔ BSP II ↔ ALP ↔ alizarin red ↔ Medium B: VEGF A ↑ Runx2 ↑ ZBTB16 ↑ NR4A3 ↑ STC1 ↑ OCN ↑ BSP II ↑ ALP ↑ alizarin red ↑	Mel alone had no significant effect on osteogenesis Osteogenesis was significantly and synergistically improved by the combination of Mel with HA + BU + RA	No odontogenic differentiation
Liu et al. (2017)	hDPSCs from 3 patients (18–25 years)	Mel 10^−12^, 10^−10^, 10^−8^ M; 1, 2, 3, 4, 5 days Mel added to osteogenic and basal growth medium	The effect of Mel was time‐ and concentration‐dependent Mel (10^−8^ M) in basal growth medium: ALP activity ↔ ALP mRNA ↔ DSPP ↔ DSPP mRNA ↔ mineralised nodules formation ↔ Mel (10^−8^ M) in osteogenic medium: ALP activity ↑ ALP mRNA ↑ DSPP ↑ DSPP mRNA ↔ mineralised nodules formation ↑	In osteogenic medium, Mel inhibited the proliferation of hDPSCs In osteogenic medium, Mel promoted odontogenic differentiation of hDPSCs	Odontogenic differentiation
Otero et al. (2017)	hDPSCs from 35 patients (18–30 years)	Mel 50 μmol; 14, 21 days	Runx2 mRNA ↔ Col 1 mRNA ↔ OP mRNA ↔ ↑ OPG mRNA ↔	Mel had no effect on the odontogenic differentiation of hDPSCs	No odontogenic differentiation
Li et al. (2020)	hDPSCs from patients (18–25 years); number of patients not given	Mel 10^−12^, 10^−10^, 10^−8^, 10^−6^, 10^−4^ M; 1, 3, 5, 7 days Mel added to the osteogenic and basal growth medium	Effects of Mel were superior in osteogenic medium Mel had no inhibitory effect on cell proliferation Mel (10^−4^ M) in osteogenic medium: DSPP mRNA ↑ DMP1 mRNA ↑ mineralised tissue formation ↑ global methylation level ↓ DNMT1 ↔ MeCP2 ↓	In osteogenic medium, Mel promoted odontogenic differentiation of hDPSCs by suppression of DNA methylation	Odontogenic differentiation
García‐Bernal et al. (2021)	hDPSCs from 10 patients; age of patients not given	Mel 3, 5, 10, 50, 100, 300, 500 μM; 96 h	Cell viability: no effect, independent of Mel concentration Cell proliferation: 24, 48, 72 h ↔ 96 h ↑ (for Mel 50, 100, 300 μM) Cell migration for Mel 10, 50, 100 μM: cell migration ↑ (after 24 and 48 h) time for covering 50% of open wound ↓ Secretion of anti‐inflammatory molecules: IL‐10 ↔ IDO ↔ PGE_2_ ↔ TGFβ ↑ Odontogenic marker expression: ALP ↔ COL1A1 ↔ ONN ↔ DSPP ↓ Runx2 ↓	Mel did not alter viability of hDPSCs Mel improved cell migration and proliferation Mel did not induce osteogenic differentiation of hDPSCs	Migration and proliferation‐promoting no odontogenic differentiation
Mancinelli et al. (2021)	hDPSCs number of patients and age not given	Equine bone blocks coated with ammonia‐functionalised graphene‐oxide (G‐N) + Mel 100 μM; 7, 14, 21 days Differentiation (DM) and basal growth medium (GM)	Cell viability (24 h): no effect of Mel Gene expression in GM: Runx2 ↑ (7 days); ↓ (14 days); ↑ (21 days) Smad5 ↑ (7 days); ↔ (14 days); ↑ (21 days) miRNAs: miR‐133a ↓ (7, 14, 21 days) miR‐133b ↓ (7 days); ↔ (14 days); ↓ (14 days) miR135a ↓ (7 days); ↔ (14 days) mir‐let‐7b ↑ (7 days) Gene expression in DM: Runx2 ↔ (7 days); ↑ (14 days); ↓ (21 days) Smad5 ↓ (7 days); ↑ (14 days); ↓ (21 days) miRNAs: miR‐133a ↓ (7, 14, 21 days) miR‐133b ↓ (7 days); ↔ (14 days); ↓ (14 days) miR135a ↓ (7 days); ↓ (14 days) mir‐let‐7b ↑ (7 days) Osteocalcin levels (DM & GM) ↑ (14 days); ↔ (21 days)	MicroRNAs have a pivotal role in the differentiation of hDPSCs Equine bone blocks and Mel have a positive effect on hDPSCs differentiation	Odontogenic differentiation
Patil et al. (2022)	hDPSCs from 12 patients (14–25 years)	Cytotoxicity test: Mel 0.5, 1, 2.5, 5, 10, 25, 50, 100 μM; 24, 48, 72 h Differentiation capacity: Mel 1, 25 μM; 1, 3, 5, 7, 9, 11, 13 days	Cytotoxicity test: Mel did not cause dose dependent influence on proliferative activity Differentiation capacity: Mel 1 μM vs. control: Runx2 ↑ COL1A1 ↑ ALP ↑ OCN ↑ OP ↑ Mel 25 μM vs. control: Runx2 ↑ COL1A1 ↑ ALP ↑ OCN ↑ OP ↔	Mel was well tolerated by hDPSCs at low (1 μM) and high concentrations (25 μM) Mel stimulated hDPSCs to differentiate into osteocytes	Odontogenic differentiation
Tumedei et al. (2022)	hDPSCs from 10 patients; age of patients not given	Mel 100 μM; 7, 14, 21 days Differentiation (DM) and basal growth medium (GM)	Gene expression in GM: Runx2 ↑ (7 days); ↔ (14, 21 days) Smad5 ↔ (7, 14, 21 days) Col5 ↓ (7 days); ↔ (14, 21 days) HDAC4 ↔ (7 days); ↑ (14 days); ↔ (21 days) miRNAs: miR‐133a ↓ (7 days); ↔ (14, 21 days) miR‐133b ↔ (7, 14, 21 days) miR135a ↔ (7, 14, 21 days) miR‐29b ↔ (7, 14, 21 days) mir‐let‐7b ↔ (7, 14, 21 days) Gene expression in DM: Runx2 ↑ (7, 14 days); ↔ (21 days) Smad5 ↑ (7, 14, 21 days) Col5 ↓ (7, 14, 21 days) HDAC4 ↓ (7, 14, 21 days) miRNAs: miR‐133a ↓ (7 days); ↔ (14, 21 days) miR‐133b ↓ (7 days); ↔ (14, 21 days) miR135a ↔ (7, 14 days); ↓ (21 days) miR‐29b ↑ (7, 14, 21 days) mir‐let‐7b ↔ (7, 14, 21 days) Osteocalcin levels: GM ↔ (14 days); ↑ (21 days) DM ↑ (14, 21 days)	Mel increased the osteogenic potential of hDPSCs hDPSCs cultured in differentiation medium showed higher osteogenic potential compared to growth medium	Odontogenic differentiation
Atila et al. (2023)	hDPSCs number of patients and age not given	Methacrylated gelatin/thiolated pectin hydrogels loaded with Mel 50 and 150 μg/mL; 1, 4, 7, 14 days	Cell viability: no effect of Mel Intracellular calcium deposition (ICD) & ALP activity: ALP (50 μg/mL) ↑ (days 7, 14) ALP (150 μg/mL) ↑↑ (day 7) ICD (50 & 150 μg/mL) ↑ (days 4, 14) Gene expression (Mel 50 & 150 μg/mL): DMP1 ↑ DSPP ↑ Axin‐2 ↑	Mel possessed stimulatory effect on odontoblastic differentiation of hDPSCs The injectable hydrogel formulation containing melatonin/tideglusib‐loaded core/shell PMMA/silk fibroin electrospun fibres can mimic extracellular matrix and the controlled release of Mel may promote vital pulp regeneration	Odontogenic differentiation
Keskin et al. (2023)	DPSCs obtained from the American Culture Collection	Mel 10^−10^, 10^−7^, 10^−4^ M; 72 h	Evaluation of cytotoxic effects of Mel on DPSCs Cell index values compared to control: Mel 10^−4^ M ↓ Mel 10^−7^ M ↔ Mel 10^−10^ M ↑	Mel increased cell proliferation at lower doses but induced cytotoxicity at higher doses	Dose‐dependent proliferation‐promoting
Zhang et al. (2024)	hDPSCs from patients (15–25 years); number of patients not given	Mel 0.1, 10 μM; 72 h	Mel 10 μM reduced viability of P7 and P12 hDPSCs; highest viability for Mel 0.1 μM Effect of Mel 0.1 μM on P7 and P12 hDPSCs: ALP staining ↑ SA‐β‐gal positive cells ↓	hDPSC senescence due to vitro expansion resulted in a decrease of osteogenic differentiation ability of hDPSCs and an increase of SA‐β‐gal positive cells Mel attenuated hDPSC senescence caused by long‐term expansion	Odontogenic differentiation Protection of hDPSC from senescence

Abbreviations: ALP, alkaline phosphatase; Axin‐2, axis inhibition protein 2; BSP, bone sialoprotein; BU, butyric acid; Col 1, collagen type 1; Col 5, collagen type 5; COL1A1, collagen type1 A1; DMP1, dentine matrix acidic phosphoprotein 1; DMPM1, dentine matrix protein‐1; DNMT1, DNA methyltransferase enzyme 1; DPSC, dental pulp stem cells; DSPP, dentine sialoprotein; HA, hyaluronic acid; HDAC4, histone deacetylase 4; hDPSC, human dental pulp stem cell; IDO, indoleamine 2,3‐dioxygenase; IL 10, interleukin 10; MeCP2, methyl‐CpG binding protein 2; Mel, melatonin; miRNA, micro ribonucleic acid; NR4A3, nuclear receptor subfamily 4, group A, member 3; OCN, osteocalcin; ONN, osteonectin; OP, osteopontin; OPG, osteoprotegerin; P7/P12, passage 7/passage 12; PGE_2_, prostaglandin E_2_; RA, retinoic acid; Runx2, runt‐related transcription factor 2; Smad5, SMAD protein 5 (S, ‘small’ worm phenotype; MAD, ‘Mothers Against Decapentaplegic’); STC1, stanniocalcin 1; TGFβ, transforming growth factor beta; VEGF A, vascular endothelial growth factor A; ZBTB16, zinc finger and BTB domain containing protein 16.

Melatonin exerted no adverse effects on cell proliferation (Li et al. 2020; Patil et al. 2022) and cell viability (García‐Bernal et al. 2021; Mancinelli et al. 2021; Atila et al. 2023), while 10 μM melatonin reduced the viability of passage 7 and passage 12 hDPSCs in a dose‐dependent way (Zhang et al. 2024).

Concurrently, six studies demonstrated that melatonin promoted odontogenic differentiation of hDPSCs (Liu et al. 2017; Li et al. 2020; Mancinelli et al. 2021; Patil et al. 2022; Tumedei et al. 2022; Atila et al. 2023; Zhang et al. 2024) (Figure 2), while according to three studies, melatonin did not induce odontogenic differentiation of hDPSCs (Maioli et al. 2016; Otero et al. 2017; García‐Bernal et al. 2021). The odontogenic differentiation of hDPSCs promoted by melatonin was found to be time‐ and concentration‐dependent (Liu et al. 2017). In three studies, melatonin was used in combination with other agents/materials: (i) butyric acid + hyaluronic acid + retinoic acid (Maioli et al. 2016): (ii) Equine bone blocks coated with ammonia‐functionalised graphene oxide (Mancinelli et al. 2021); (iii) melatonin/tideglusib‐loaded core/shell PMMA/silk fibroin electrospun fibres (Atila et al. 2023). While melatonin alone had no significant effect on osteogenesis, osteogenesis was significantly and synergistically improved by the combination of melatonin with butyric acid + hyaluronic acid + retinoic acid (Maioli et al. 2016). Equine bone blocks and melatonin showed a positive effect on hDPSCs differentiation (Mancinelli et al. 2021). The controlled release of melatonin from melatonin/tideglusib‐loaded core/shell PMMA/silk fibroin electrospun fibres caused a stimulatory effect on odontoblastic differentiation of hDPSCs (Atila et al. 2023).

Keskin et al. (2023) found a dose‐dependent proliferation‐promoting effect, as melatonin increased cell proliferation at lower doses but induced cytotoxicity at higher doses. Also, in another study, melatonin improved cell migration and proliferation (García‐Bernal et al. 2021). Moreover, melatonin attenuated hDPSCs senescence caused by long‐term expansion (Zhang et al. 2024).

## 4 Discussion

The aim of this scoping review was to appraise the currently available evidence regarding the impact of melatonin on dental pulp tissues and dental pulp stem cells, as recently potential therapeutic effects of melatonin have gained attention in dentistry (Gómez‐Moreno et al. 2010; Blasiak et al. 2011). As during the initial literature search it became obvious very early that the currently available studies are highly heterogeneous with regard to the concentration of administered melatonin, cell types used, age and health conditions of donors as well as investigated parameters a meta‐analysis was considered inappropriate. Therefore, a scoping review instead of a systematic review with meta‐analysis was regarded as suitable to answer the formulated research questions.

### 4.1 Effect of Melatonin on Dental Pulp Tissue

The main findings were that melatonin (i) exerts anti‐inflammatory, antioxidant, anti‐fibrinolytic and anti‐apoptotic effects on dental pulp cells and tissues, (ii) has the potential to protect the dental pulp from exogenous noxa and (iii) seems to be a suitable agent for direct pulp capping (Tables 2 and 3; Figure 2). In healthy human dental pulp tissue, melatonin is expressed within the odontoblastic zone, cell‐rich zone and in the connective tissue with a strong expression of melatonin receptors 1 & 2 (MT1; MT2) (Kantrong et al. 2020). In the case of pulpitis, MT1 expression increases but expression of MT2 remains constant (Kantrong et al. 2020) and due to an activation of the TLR4/NF‐κB signalling pathway pro‐inflammatory cytokine expression is induced (Li et al. 2015).

Based on their results, Kantrong et al. (2020) speculated that MT1 has an important function in the production of reactionary dentine during pulpal inflammation, which is in agreement with a previous report (Tachibana et al. 2014). It seems that the coordination of receptor isoforms MT1 and MT2 is momentous regarding the modulation of congenital immunity within the pulp tissue and probably these two receptors have opposite functions in the pulp. It seems that for melatonin produced in response to microbial infection in the pulp, MT1 acts as a selective sensor (Kantrong et al. 2020).

When interpreting the following findings, it must be taken into consideration that melatonin production as well as MT1 expression decline with age (Martínez et al. 2025). At the age of 60 years the melatonin production is reduced by about 50% and at the age of 70 years by about 90% compared to the primary production at the age of 20 years (Martínez et al. 2025). The age‐related decline in melatonin receptor expression (MT1 & MT2) is linked to a decreased ability to respond to melatonin with age (Martínez et al. 2025). Although a specific physiological concentration of melatonin in sound dental pulp tissue has not been stated (physiological systemic concentration is in the range of 10^−12^ to 10^−8^ M), based on general melatonin physiology it is reasonable to assume that also in the dental pulp the melatonin concentration and receptor expressions decrease with age. Therefore, it can be speculated that the following described protective effects of melatonin may be less distinct in older individuals, which probably might have an adverse effect on the success rates of vital pulp therapy. However, these assumptions require further investigations.

The anti‐inflammatory effect of melatonin is based on different mechanisms (Kantrong et al. 2020). Melatonin can decrease the gene expression of the pro‐inflammatory cytokines TNF‐α and IL‐1β (Li et al. 2015; Kantrong et al. 2020; Kermeoğlu et al. 2021; Chang et al. 2022). This reduced expression of cytokines is caused by a melatonin‐induced suppression of the TLR4/NF‐ĸB signalling pathway, as the gene expression of TLR4 and NF‐ĸB was decreased (Li et al. 2015). Based on their results the authors speculated that low melatonin levels on the first day of acute pulpitis may be partially responsible for the development of acute pulpitis (Li et al. 2015).

Furthermore, melatonin may act as an antagonist against COX‐2 induction in pulpal fibroblasts (Kantrong et al. 2020). Obviously, melatonin has selective antagonistic properties against lipopolysaccharide (LPS)‐mediated pulp inflammation via COX‐2 and IL‐1β signalling (Kantrong et al. 2020). Melatonin was able to down‐regulate LPS‐induced IL‐1β expression in a dose‐dependent manner (Kantrong et al. 2020). The authors concluded that melatonin together with its receptors (MT1 and MT2) can be regarded as ‘a first line defence against microbial insult in dental pulp’ (Kantrong et al. 2020).

Chang et al. (2025) reported another signalling pathway how melatonin may control bacterial pathogen‐induced pulpal inflammation. Carious lesions, for instance, may result in the invasion of pathogenic bacteria into the pulp. A degradation product of peptidoglycans in gram‐positive and most of the gram‐negative bacteria pulpal pathogens is L‐Ala‐γ‐D‐Glu‐mDAP (TriDAP), a DAP‐comprising muramyl tripeptide. In hDPCs, TriDAP activates nucleotide‐binding oligomerization domain1/2 (NOD1/NOD2), which in turn causes activation of TAK1 (transforming growth factor‐β (TGF‐β)‐activated kinase 1), p38 (p38 mitogen‐activated protein kinases) and p‐MEK/ERK (phosphorylated mitogen‐activated protein kinases/extracellular signal‐regulated kinases) and these kinases mediate the expression of cytosolic phospholipase A2 (cPLA2) and cyclooxygenase‐2 (COX‐2), which finally results in the production of prostaglandin (PGE_2_ and PGE_2α_) production. Melatonin was found to inhibit the TriDAP‐induced prostaglandin production and the authors speculated that melatonin may become a pharmacological agent to control bacterial pathogen‐induced pulpal inflammation in the future (Chang et al. 2025).

Moreover, in the case of LPS‐induced acute pulpitis, expression and activation of proteolytic matrix metalloproteinases (MMP‐1 as a member of collagenases and MMP‐2 (gelatinase A)) were increased in rats (Kermeoğlu et al. 2021). As the systemic administration of melatonin reduced the expression of MMP‐1 and MMP‐2, melatonin was able to protect the dental pulp from pulpitis (Kermeoğlu et al. 2021).

As mentioned before, the expression of IL‐1β is increased in the case of pulpitis. This pro‐inflammatory cytokine can affect plasminogen activation system molecules, such as urokinase‐type plasminogen activator (uPA), urokinase‐type plasminogen activator receptor (uPAR) and plasminogen activator inhibitor 1 (PAI‐1) (Chang et al. 2022). The pathological process within the pulp tissue (thrombolytic and pericellular extracellular matrix degradation; breakdown of angiogenesis; cytokine production) is triggered by plasmin (Chang et al. 2022). Melatonin was found to effectively suppress uPA levels and to stimulate uPAR expression and soluble urokinase‐type plasminogen activator receptor (suPAR) production of hDPCs (Chang et al. 2022). The increased expression of uPAR and suPAR is relevant for important biological activities within the pulp regarding cell adhesion and proliferation, tissue homeostasis, blood coagulation and proteolysis (Chang et al. 2022). The authors concluded that melatonin exerts an anti‐fibrinolytic effect on IL‐1β‐induced changes and might be beneficial for the prevention and treatment of pulp inflammation (Chang et al. 2022).

Four studies investigated the antioxidant effect of melatonin with partially controversial results (Milosavljević et al. 2018; Deng et al. 2019; Barać et al. 2023; Ilić et al. 2023). In H_2_O_2_‐induced pulp inflammation melatonin failed to effectively reduce ROS and apoptosis and decreased the mitochondrial membrane potential (Δᴪm) (Deng et al. 2019), which is in disagreement with the majority of other studies that assessed the antioxidant effect and impact of melatonin on mitochondrial function in other tissues or organs (Adamiak et al. 2024; Al‐Suhaimi et al. 2024). A recent review stated that melatonin exhibits a protective role against oxidative stress and mitochondrial synthesis of ROS and can be regarded as a defence mechanism against ageing‐related diseases (Al‐Suhaimi et al. 2024). In contrast to the results reported by Deng et al. (2019), three other studies using hDPCs from type 2 diabetic donors found that under hyperglycaemic conditions, melatonin normalised inducible NO synthase (iNOS) and superoxide dismutase (SOD) activity levels (Milosavljević et al. 2018; Barać et al. 2023; Ilić et al. 2023). Thus, according to the results of these studies melatonin exerts an antioxidant and protective effect on dental pulp cells. These findings are corroborated by the results of a very recent study using hDPSCs (Peng et al. 2025). Melatonin significantly increased the stress resistance of cell mitochondria and maintained hDPSCs cellular homeostasis to adapt to oxidative stress. Nicotinamide adenine dinucleotide (NAD^+^) metabolism was rebalanced by melatonin and mitochondrial and cellular ROS levels were reduced (Peng et al. 2025). The authors concluded that for the protective effect of melatonin the NAD^+^‐dependent signalling may be a promising strategy (Peng et al. 2025). However, in summary, the antioxidant effects of melatonin and its impact on mitochondrial functions in the dental pulp are not yet fully explored and further investigations are needed.

In pulp tissue obtained from type 2 diabetic patients, expression of melatonin was significantly reduced (Milosavljević et al. 2018; Barać et al. 2023; Ilić et al. 2023). Diabetes induces oxidative‐nitrosative stress in diabetic pulp tissue, as iNOS and SOD activity, and glial cell line‐derived neurotropic factor (GDNF) were markedly increased compared with normoglycemic levels (Milosavljević et al. 2018; Barać et al. 2023; Ilić et al. 2023). iNOS and p300 protein expression levels showed a strong positive correlation under hyperglycaemia, which suggests a possible involvement of histone acetyltransferase p300 (p300) in the hyperglycaemia‐induced increase of iNOS levels (Milosavljević et al. 2018). This could be of relevance as the p300/NF‐ĸB signalling pathway plays a crucial role in the pathogenesis of chronic diabetes complications (Chiu et al. 2009). Consistently, three studies found that under hyperglycaemic conditions, melatonin has antioxidant and protective effects on pulp cells due to its ability to normalise iNOS and SOD activity levels (Milosavljević et al. 2018; Barać et al. 2023; Ilić et al. 2023) and to reduce GDNF (Barać et al. 2023). In diabetic tissues, up‐regulation of GDNF may enhance protein glycosylation but is also linked to oxidative stress (Reily et al. 2019). In summary, the obtained results contribute to the understanding of diabetes‐associated disturbances in dental pulp tissue (Lima et al. 2013; Gonzalez Marrero et al. 2022) and obviously melatonin is able to induce stress‐protective mechanisms in hyperglycaemic hDPCs (Barać et al. 2023).

One animal study evaluated the suitability of melatonin as an agent for direct pulp capping in comparison with a calcium silicate‐based cement (MTA) (Guerrero‐Gironés et al. 2020). Three melatonin groups were established: 5 mg topical application, MTA + melatonin 10 mg/100 mL administered orally and melatonin 5 and 245 mg cornstarch in distilled water (topical) + melatonin 10 mg/100 mL administered orally. Regarding the degree of pulp inflammation or pulp necrosis, dentinal bridge formation, structure of the odontoblastic layer and extent of pulp fibrosis no significant differences between MTA and the melatonin groups were observed. However, the results of this study should be interpreted with care as cavity depths and sizes were not standardised, resulting in varying degrees of pulpal injury.

Furthermore, melatonin had no effect on the basal level of oxidative stress. These findings regarding dentinal bridge formation with melatonin can be explained by the ability of melatonin to stimulate osteoblast differentiation through bone morphogenetic proteins (BMP‐2, BMP‐4), and growth factors and to reduce the time required for the differentiation of osteoblasts from 21 to 12 days (Park et al. 2011; Guerrero‐Gironés et al. 2020). The melatonin 1a receptor (Mel1aR) seems to be responsible for dental hard tissue formation caused by melatonin (Tachibana et al. 2014).

Three studies showed that melatonin has a protective effect against exogenous noxa (Doğan et al. 2017; Ilić et al. 2023; Yu et al. 2024). ELF‐EMF caused odontoblast degeneration, increased inflammatory cell infiltration, dilatation in blood vessels and haemorrhage in the pulp tissue of Wistar rats and oral administration of melatonin (10 mg/kg/day) increased the activity of odontoblasts and induced the proliferation of fibroblasts and neo‐angiogenesis (Doğan et al. 2017). After 26 and 52 days, vascular Endothelial Growth Factor (VEGF) positive expression was observed in the blood vessels close to the odontoblastic layer (Doğan et al. 2017). A recent review concluded that melatonin can protect the pulp tissue from the adverse effects of monomers derived from resin‐based restorations (Blasiak et al. 2011). This was confirmed, as Ilić et al. (2023) found that melatonin counteracted iNOS‐mediated pulp inflammation and decreased the SOD activity in HEMA (2‐hydroxylmethyl methacrylate)‐treated hDPCs. Furthermore, Yu et al. (2024) reported that in mouse preodontoblast cells melatonin reduced mitochondrial reactive oxygen species (mtROS), mitochondrial membrane potential (MMP) and adenosine triphosphate (ATP) levels. Thereby, melatonin was able to antagonise TEGDMA (triethylene glycol dimethacrylate)‐induced cell apoptosis. The authors concluded that melatonin plays an important role in attenuating mitochondrial dysfunction‐regulated apoptosis through the JNK/MAPK signalling pathway (c‐Jun N‐terminal kinase/mitogen‐activated protein kinase) as TEGDMA causes mitochondrial oxidative damage via JNK‐dependent autophagy (Yu et al. 2024).

Regarding the findings obtained from animal studies using rat incisors or molars (Table 3), extrapolating the results to human pulp tissue should be done with the utmost care. Although a review pointed out that rat molar teeth are a suitable study model regarding the assessment of pulp tissue reactions (Dammaschke 2010), the exceptional resilience and healing capacity of rat molar pulps must be considered (Goldberg and Smith 2004). Moreover, the pulp tissue of rats is more reactive compared to human pulp and rat pulps are less susceptible to postoperative infection due to specific immune defence mechanisms (overview by Dammaschke 2010). Another crucial aspect that limits the transferability of the results obtained from rodent studies to human pulp is the use of rat incisors in two included studies (Doğan et al. 2017; Kermeoğlu et al. 2021). In contrast to human teeth, rat incisors are continuously growing teeth; therefore, extrapolating such findings to human pulp appears to be problematic. Hence, against this background future studies investigating the effects of melatonin on dental pulp tissue should use pulp cells and tissues obtained from human donors instead of using a rodent model.

Finally, as melatonin is a lipophilic and poorly water‐soluble molecule it is noteworthy that detailed information on how it was dissolved for administration is not mentioned in all included studies. Moreover, neither solvents used nor vehicle controls are consistently reported. Solvents that are reported include a 5% ethanol solution in saline without solvent controls (Li et al. 2015), 100% ethanol without solvent controls (Mancinelli et al. 2021) and 96% ethanol with solvent‐treated controls (Milosavljević et al. 2018). Thus, some uncertainty regarding the potential side effects of solvents or vehicles used remains and future studies should take these aspects into account by providing detailed information on solvents or vehicles used and by establishing appropriate control groups.

### 4.2 Effect of Melatonin on Human Dental Pulp Stem Cells (hDPSCs)

The available results regarding the effect of melatonin on proliferation, viability and migration of hDPSCs are not fully consistent. Some studies reported that melatonin did not show any dose‐dependent influence on proliferative activity (Li et al. 2020; Patil et al. 2022) or cell viability (García‐Bernal et al. 2021; Mancinelli et al. 2021; Atila et al. 2023). Contrarily, other studies found a time‐ and/or dose‐dependent effect of melatonin on cell proliferation (García‐Bernal et al. 2021; Keskin et al. 2023) and cell migration (García‐Bernal et al. 2021). At lower doses (10^−10^ M) melatonin increased cell proliferation, but induced cytotoxicity at higher doses (10^−4^ M) (Keskin et al. 2023). These findings are in agreement with those of Liu et al. (2017), as a time‐ and concentration‐dependent effect of melatonin was reported. With elevated doses (from 10^−12^ to 10^−8^ M) of melatonin the cytotoxic effect increased (Liu et al. 2017), while according to the findings of García‐Bernal et al. (2021) no effect of melatonin on cell proliferation was found after 24, 48 and 72 h, but after 96 h melatonin improved cell proliferation significantly. This effect was found to be also dose‐dependent, with superior results for intermediate concentration (10–300 μM) (Table 4). While low‐dose melatonin (0.1 μM) exerted no significant effect on cell viability, higher concentrations (10 μM) inhibited cell activity, especially in passage 7 cells (Zhang et al. 2024). Moreover, melatonin exerted a dose‐ and time‐dependent effect on migration of hDPSCs after 24 and 48 h and the wound healing assay revealed that melatonin significantly reduced the time required for covering 50% of the open wound (García‐Bernal et al. 2021). The author postulated that these effects may be due to MT2 signalling with downstream phosphorylation of focal adhesion kinase and stimulation of cytoskeletal proteins (e.g., profilin 1, F‐actin) (Lee et al. 2014). However, this explanation remains questionable, as another study did not detect melatonin receptors in hDPSCs (Liu et al. 2017). On the whole, available results suggest that different concentrations of melatonin can trigger different biological effects (Zhang et al. 2024). This hypothesis warrants further investigations.

With regard to in vitro expansion of hDPSCs for therapeutical aspects, it might be of relevance that melatonin can protect hDPSCs from senescence during long‐term expansion (Zhang et al. 2024). It is known that the matrix metalloproteinase 3 (MMP‐3) has a crucial role in cell senescence (Birch and Gil 2020) and melatonin seems to be able to downregulate MMP‐3 via IL6‐JAK‐STAT3, NF‐κB/TNF‐α signalling (Zhang et al. 2024). Melatonin reduces the production of MMP‐3 by inhibiting Sirtuin‐1‐dependent nicotinamide phosphoribosyltransferase (NAMPT) and nuclear factor of activated T‐cells 5 (NFAT5) signalling (Guo et al. 2017) and by binding to the MMP‐3 active site directly (Choudhary et al. 2022). The melatonin‐induced protection of hDPSCs from senescence was corroborated in a recent study (Peng et al. 2025). Melatonin attenuated cell senescence and apoptosis by rebalancing nicotinamide adenine dinucleotide (NAD^+^) metabolism, reducing mitochondrial and cellular ROS levels and diminishing mitochondrial dysfunction (Peng et al. 2025).

Regarding the studies focussing on odontogenic differentiation of hDPSCs, two studies failed to show that melatonin induced or accelerated differentiation (Otero et al. 2017; García‐Bernal et al. 2021), neither at transcript levels of osteogenic‐specific markers (e.g., runt‐related transcription factor 2 (Runx2), collagen type 1 (Col 1), osteonectin (OCN), osteopontin (OP), osteoprotegerin (OPG)) (Otero et al. 2017; García‐Bernal et al. 2021) nor regarding mineralised nodule formation (García‐Bernal et al. 2021). Interestingly, another study reported that melatonin alone had no significant effect on osteogenesis, but osteogenesis was significantly and synergistically improved by the combination of melatonin with butyric acid + hyaluronic acid + retinoic acid (Maioli et al. 2016). The addition of the three acids resulted in increased expression of vascular endothelial growth factor A (VEGF A), which mediates bone vascularisation and differentiation of cells towards osteoblasts, and zinc finger and BTB domain containing protein 16 (ZBTB16) and nuclear receptor subfamily 4, group A, member 3 (NR4A3). Both ZBTB16 and NR4A3 play an important role in stem cell osteogenesis and ZBTB16 is able to organise an osteogenic program independently of Runx2 (Maioli et al. 2016).

According to the results of six studies, melatonin promoted odontogenic differentiation of hDPSCs (Liu et al. 2017; Li et al. 2020; Mancinelli et al. 2021; Patil et al. 2022; Tumedei et al. 2022; Atila et al. 2023; Zhang et al. 2024). It is noteworthy that the mechanisms underlying the effects of melatonin on hDPSCs seem to be mediated by non‐receptor‐dependent pathways, as Liu et al. (2017) were unable to detect melatonin receptors in hDPSCs. Thus, regulation of cell proliferation and differentiation by melatonin seems to be based on other signalling pathways. Obviously, melatonin can act on several genes in hDPSCs, driving them towards osteogenic lineage (Patil et al. 2022; Atila et al. 2023). For instance, melatonin showed a dose‐dependent effect on Runx2, OCN, collagen type1 A1 (COL1A1) and OPN gene expression (Patil et al. 2022). When using a hydrogel formulation containing melatonin, upregulated gene expression of osteogenesis markers, namely axis inhibition protein 2 (Axin‐2), dentine sialoprotein (DSPP) and dentine matrix acidic phosphoprotein 1 (DMP1) was found (Atila et al. 2023).

An impact of melatonin on gene expression of alkaline phosphatase (ALP), Smad5 (SMAD protein 5 (S = ‘small’ worm phenotype; MAD = ‘Mothers Against Decapentaplegic’)) and OPN as early markers of osteogenesis and on Runx2, OCN, COL1A1 and bone sialoprotein (BSP) as late markers of osteogenesis was also found in other studies (Mancinelli et al. 2021; Tumedei et al. 2022). The authors of the latter study assumed that the differentiation process is controlled through the melatonin‐dependent activation of Runx2 and Smad5 (Mancinelli et al. 2021; Tumedei et al. 2022). This assumption is based on investigations on micro ribonucleic acids (miRNA), which are short, non‐coding RNAs modulating gene expression at the post‐transcriptional level. miRNAs are mediators of osteoblast differentiation and can regulate several signalling pathways, such as the Runx2 expression, and miR‐133 and miR‐135 inhibit stem cell differentiation (Tumedei et al. 2022). In differentiation medium, melatonin downregulated miR‐133a, miR‐133b and miR‐135a but upregulated Runx2 and Smad5 activity (Tumedei et al. 2022). These findings are in line with those reported in another study by the same research group, as again melatonin downregulated the gene expression of miR‐133a, miR‐133b and miR‐135a (Mancinelli et al. 2021). Furthermore, the expression of miR‐let‐7, which can promote osteogenic differentiation, was investigated. Melatonin significantly upregulated miR‐let‐7, which might confirm that miR‐let‐7 can enhance the differentiation process of hDPSCs (Mancinelli et al. 2021). Thus, by regulating the expression of transcription factors relevant for odontogenic differentiation, miRNAs play a crucial role in the differentiation of dental pulp stem cells.

Li et al. (2020) studied another pathway possibly involved in the differentiation of hDPSCs, DNA methylation. DNA methylation is operated by DNA methyltransferases (DNMTs; e.g., DNMT1) and methyl‐CpG‐binding domain proteins (MBDs; e.g., methyl‐CpG binding protein 2 (MeCP2)). It is known that hypo‐methylation promotes stem cell differentiation (Berdasco et al. 2012) and that the DNA demethylation machinery has a direct impact on the differentiation potential of hDPSCs (Li et al. 2018). Furthermore, in goat oocytes, melatonin promoted differentiation by downregulating the expression of DNMTs and reducing the global methylation level (Saeedabadi et al. 2018). Li et al. (2020) using hDPSCs, showed that melatonin regulated the levels of DNMT1 and downregulated MeCP2 levels and the global methylation level. The authors postulated that melatonin has an effect on MeCP2 or the MeCP2‐DNMT1 complex and that the melatonin‐induced suppression of DNA methylation promotes the differentiation of hDPSCs.

Besides these results gained from evaluations of gene expression of osteogenic markers, the impact of melatonin on hDPSCs differentiation was also confirmed by assessment of mineralised nodule formation, intracellular calcium deposition and ALP (alkaline phosphatase) activity (Maioli et al. 2016; Liu et al. 2017; Patil et al. 2022; Atila et al. 2023; Zhang et al. 2024). Furthermore, osteocalcin levels were determined in some studies (Mancinelli et al. 2021; Patil et al. 2022; Tumedei et al. 2022). Pretreatment of hDPSCs resulted in increased ALP activity (Liu et al. 2017; Patil et al. 2022; Atila et al. 2023; Zhang et al. 2024) and osteocalcin levels in differentiation medium (Mancinelli et al. 2021; Patil et al. 2022; Tumedei et al. 2022). Using different staining methods, melatonin‐induced mineralised tissue formation and intracellular calcium deposition were found in two studies (Li et al. 2020; Atila et al. 2023).

In summary, the limited evidence available suggests that melatonin might have the potential as a stem cell modulator (Figure 2).

It is worth mentioning that hDPSCs have been shown to possess a marked clinical potential in regenerative medicine for various diseases (Yamada et al. 2019). An elaborated review pointed out that according to the present knowledge, hDPSCs are effective for Alzheimer's disease, cerebral ischaemia, diabetes, eye disease, immune disease, liver cirrhosis, myocardial infarction, muscular dystrophy, spinal cord injuries and Parkinson's disease (Yamada et al. 2019). In concordance with the results presented above, melatonin has been shown to exert a promising and important role regarding the function of mesenchymal stem cells in medical disease therapy (Zhang et al. 2017). It is able to promote proliferation, migration and differentiation of stem cells in bone, brain, kidney, lung, nervous system, liver, heart and bladder diseases (Zhang et al. 2017) and in skin wound healing (Adamiak et al. 2024). Melatonin‐preconditioned hDPSCs (1, 10 and 100 μM melatonin) showed improved bone regeneration in calvarial bone defects (Chan et al. 2022). Moreover, melatonin (0.01, 0.1, 1 and 10 μM) promoted the hepatic differentiation of hDPSCs and was therefore regarded as a promising approach for the treatment of liver cirrhosis (Cho et al. 2015). In an animal model of spinal cord injury, melatonin (5 μM) preconditioning on hDPSCs resulted in significantly improved neuronal differentiation and engraftment compared to the use of non‐pretreated hDPSCs (Naeimi et al. 2024). Similarly, melatonin‐induced differentiation of hDPSCs into neuronal‐like cells has recently been shown (Baysal et al. 2021). On the whole, the available results from regenerative medicine are in good agreement with those obtained in endodontics (Table 4).

Although the effects of melatonin on periapical lesions and stem cells from the apical papilla were beyond the scope of this review, it is notable that a similar effect as described in this scoping review was reported. In brief, due to its anti‐inflammatory and antioxidant activity, melatonin showed antiresorptive effects on experimentally induced periapical lesions in rats (Sarıtekin et al. 2019; Dos Santos et al. 2023; Kırmızı et al. 2024). Also in rats, systemic administration of melatonin (10 mg/kg/day) resulted in improved tissue response to three different endodontic sealers by modulating the inflammatory and reparative process (de Oliveira et al. 2024). In a dose‐dependent effect on cell viability, melatonin enhanced osteogenic/odontogenic differentiation of human stem cells from apical papilla (Karkehabadi et al. 2023).

### 4.3 Clinical Relevance

Based on the anti‐inflammatory, antioxidant, anti‐fibrinolytic and anti‐apoptotic effects of melatonin on dental pulp cells and tissues, this agent might have the potential to suppress bacterial‐induced pulpal inflammation in the early stage of acute pulpitis (Chang et al. 2025). Both topical and systemic administration of melatonin seems to be suitable for pulp protection. Especially in diabetic patients, melatonin may become a protective pharmacological agent to shelter the pulp tissue from diabetes‐associated disturbances (Milosavljević et al. 2018; Barać et al. 2023; Ilić et al. 2023). However, the effect of melatonin on insulin secretion must be considered as melatonin inhibits insulin secretion and can result in higher blood glucose levels and an increased risk for type 2 diabetes (Peschke et al. 2007). This interrelation requires further clinical studies.

Regarding systemic administrations, potential adverse effects associated with currently available melatonin formulations, such as drowsiness, hormonal interactions and dose‐dependent variability, must be considered (Martínez et al. 2025). Therefore, any systemic application for pulp protection should be approached with caution, and further preclinical safety studies are mandatory.

Moreover, melatonin was found to be a suitable agent for direct pulp capping in comparison with MTA (Guerrero‐Gironés et al. 2020). However, as melatonin failed to cause better results than the current gold standard for vital pulp treatment (calcium silicate‐based materials) and the fact that overwhelming and sound evidence regarding the clinical outcome of vital pulp treatment using calcium silicate‐based materials is available (Cushley et al. 2021; Komora et al. 2024), the use of melatonin as an agent for vital pulp treatment seems to be of minor relevance from a clinical point of view.

As melatonin might have the potential as a stem cell modulator (Table 4), injectable hydrogel formulations containing melatonin‐loaded fibres can mimic the extracellular matrix, and the controlled release of melatonin may promote vital pulp regeneration (Atila et al. 2023). This finding and the observation that in a dose‐dependent way, melatonin can enhance osteogenic/odontogenic differentiation of hDPSCs (Karkehabadi et al. 2023) may offer new concepts in both cell‐based and cell‐free endodontic tissue engineering. From a clinical point of view, this aspect is particularly interesting as according to the recent S3 Guideline of the ESE (Duncan et al. 2023), it is currently unclear whether endodontic tissue engineering represents a valid treatment option. Due to this lack of evidence, further research is necessary (Widbiller et al. 2023) and the documented properties of melatonin may be the key for future clinical studies.

### 4.4 Strengths and Limitations

The strengths of this review are that the timeframe of the literature search was extended up to the 1st of July 2025 to ensure that no new relevant studies were neglected. Furthermore, the literature search and the following data extraction were conducted by two independent reviewers, with a high inter‐reviewer reliability of 0.888. Moreover, up to now, only one narrative review summarised the results of in vivo, in vitro and clinical studies on the effects of melatonin in wound healing of dental pulp tissue (Vaseenon et al. 2021). Nine studies, published before April 2020, were included. However, since then, the available knowledge has markedly increased as several studies conducted after spring 2020 have become available. Thus, compared with this previous review, the conclusions drawn from the present scoping review, with a more systematic and detailed search process, are based on a broader foundation, as more than twice as many relevant studies were assessed.

On the other hand, the limitations of this scoping review are twofold. Firstly, all conclusions were drawn from laboratory and animal studies, as randomised controlled or at least clinical studies are not available yet. This emphasises the urgent need, in order to transfer the currently available results to clinical scenarios, to conduct well‐designed clinical studies with a sufficient sample size. Secondly, a minor limitation represents the fact that the language of the included studies was restricted to English, which holds the risk of a potential language bias.

## 5 Conclusions

The limited evidence from laboratory and animal studies suggests that

- melatonin shows anti‐inflammatory, antioxidant and anti‐fibrinolytic effects on dental pulp tissue;
- melatonin can protect the dental pulp against exogenous noxa;
- melatonin may have anti‐apoptotic effects on preodontoblast cells and seems to protect dental pulp stem cells from senescence;
- melatonin has potential as a stem cell modulator and may promote odontogenic differentiation of dental pulp stem cells;
- melatonin may improve migration and proliferation of dental pulp stem cells;
- some findings point towards a dose‐ and time‐dependent effect of melatonin, but this scoping review fails to extract reliable data regarding the most efficient concentration of melatonin.

## Author Contributions

J.S.: literature search, data extraction, writing; K.K.: writing, editing; E.S.: literature search, data extraction, writing, supervision.

## Funding

The authors have nothing to report.

## Conflicts of Interest

The authors declare no conflicts of interest.

## Data Availability

The data that support the findings of this study are available on request from the corresponding author. The data are not publicly available due to privacy or ethical restrictions.
